# Oxytocin Is a Positive Allosteric Modulator of κ-Opioid Receptors but Not δ-Opioid Receptors in the G Protein Signaling Pathway

**DOI:** 10.3390/cells10102651

**Published:** 2021-10-04

**Authors:** Kanako Miyano, Yuki Yoshida, Shigeto Hirayama, Hideki Takahashi, Haruka Ono, Yoshiyuki Meguro, Sei Manabe, Akane Komatsu, Miki Nonaka, Takaaki Mizuguchi, Hideaki Fujii, Yoshikazu Higami, Minoru Narita, Yasuhito Uezono

**Affiliations:** 1 Division of Cancer Pathophysiology, National Cancer Center Research Institute, Tokyo 104-0045, Japan; kmiyano@ncc.go.jp (K.M.); minarita@ncc.go.jp (M.N.); 2 Department of Pain Control Research, The Jikei University School of Medicine, Tokyo 105-8461, Japan; a-komats@juntendo.ac.jp (A.K.); minonaka@jikei.ac.jp (M.N.); 3 Department of Medicinal and Life Sciences, Faculty of Pharmaceutical Sciences, Tokyo University of Science, Chiba 278-8510, Japan; groadmlargo@gmail.com (Y.Y.); higami@rs.tus.ac.jp (Y.H.); 4 Department of Medicinal Chemistry, School of Pharmacy, Kitasato University, Tokyo 108-8641, Japan; hirayamas@pharm.kitasato-u.ac.jp (S.H.); pp14145@st.kitasato-u.ac.jp (H.T.); pp15061@st.kitasato-u.ac.jp (H.O.); mizuguchit@pharm.kitasato-u.ac.jp (T.M.); fujiih@pharm.kitasato-u.ac.jp (H.F.); 5 Department of Surgery, Division of Gastroenterological, General and Transplant Surgery, Jichi Medical University School of Medicine, Tochigi 329-0498, Japan; y-meguro@jichi.ac.jp; 6 Department of Anesthesiology and Resuscitology, Okayama University Graduate School of Medicine, Dentistry and Pharmaceutical Sciences, Okayama 700-8558, Japan; me421081@s.okayama-u.ac.jp; 7 Department of Anesthesiology and Pain Medicine, Juntendo University Graduate School of Medicine, Tokyo 113-8421, Japan; 8 Department of Pharmacology, Hoshi University School of Pharmacy and Pharmaceutical Sciences, Tokyo 142-8501, Japan

**Keywords:** oxytocin, kappa-opioid receptor, positive allosteric modulator, opioids, G-protein signaling pathway, opioid receptor

## Abstract

Oxytocin (OT) influences various physiological functions such as uterine contractions, maternal/social behavior, and analgesia. Opioid signaling pathways are involved in one of the analgesic mechanisms of OT. We previously showed that OT acts as a positive allosteric modulator (PAM) and enhances μ-opioid receptor (MOR) activity. In this study, which focused on other opioid receptor (OR) subtypes, we investigated whether OT influences opioid signaling pathways as a PAM for δ-OR (DOR) or κ-OR (KOR) using human embryonic kidney-293 cells expressing human DOR or KOR, respectively. The CellKey^TM^ results showed that OT enhanced impedance induced by endogenous/exogenous KOR agonists on KOR-expressing cells. OT did not affect DOR activity induced by endogenous/exogenous DOR agonists. OT potentiated the KOR agonist-induced Gi/o protein-mediated decrease in intracellular cAMP, but did not affect the increase in KOR internalization caused by the KOR agonists dynorphin A and (-)-U-50488 hydrochloride (U50488). OT did not bind to KOR orthosteric binding sites and did not affect the binding affinities of dynorphin A and U50488 for KOR. These results suggest that OT is a PAM of KOR and MOR and enhances G protein signaling without affecting β-arrestin signaling. Thus, OT has potential as a specific signaling-biased PAM of KOR.

## 1. Introduction

Opioid receptors (ORs), which belong to the class A family of G protein-coupled receptors (GPCRs), are mainly classified into three different types: μ-OR (MOR), δ-OR (DOR), and κ-OR (KOR) [1,2,3]. In addition to these three major receptors, the nociception/orphanin FR receptor was identified as the fourth member of the OR family [4]. Moreover, other opioid receptors, such as ζ-, ε-, λ-, and ι-ORs, have also been characterized [5]. The three classical ORs (MOR, DOR, and KOR) are primarily expressed in the spinal cord, brain stem, thalamus, and cortex, which together constitute the ascending pain transmission system and the descending inhibitory pain system [6]. Activation of these ORs decreases presynaptic transmitter release, hyperpolarization of postsynaptic elements, and disinhibition^6^. Endogenous opioid peptides, such as β-endorphin and dynorphin A, are related to the endogenous pain modulatory system [6]. Traditional opioid drugs, such as morphine, are used for acute and chronic cancer pain. However, these drugs often cause adverse effects, including respiratory depression, dependency, and tolerance; therefore, in-depth research has been performed on opioid cell signaling to overcome these adverse effects [7,8]. The activation of these receptors induces two of the most important GPCR signaling pathways: the G protein-dependent signaling pathway and the β-arrestin signaling pathway. G protein-dependent signaling pathways involve inhibition of adenylyl cyclase and activation of G protein-coupled inwardly rectifying K^+^ channels via G_i_/G_o_ proteins, which are supposed to induce pain relief. The β-arrestin-dependent signaling pathway involves OR internalization via β-arrestin recruitment to ORs; this pathway is involved in adverse effects such as respiratory depression and tolerance.

To overcome these adverse effects of the existing opioid drugs, research on G protein-biased agonists or positive allosteric modulators (PAMs) is being conducted to develop novel opioid drugs. G protein-biased agonists activate G protein-dependent signaling, but have a lesser effect on β-arrestin-dependent signaling. Several G protein-biased agonists such as TRV130 or PMZ21 have been discovered for MOR [9,10]; TRV130 (also known as oliceridine) was approved by the US FDA in 2020 and has a lower incidence of adverse events (respiratory depression and constipation) than equianalgesic morphine [11]. In addition, G protein-biased DOR and KOR agonists have also been synthesized and are expected to be analgesics with fewer adverse effects [12,13].

PAMs bind to an allosteric site on the receptor, which is separate from the orthosteric site that binds an endogenous agonist, and then potentiates the affinity and/or efficacy of orthosteric agonists [14]. To date, many GPCRs have been found to be PAMs [15,16]. In the case of ORs, PAMs for MOR and for both MOR and DOR were identified in the 2010s [16,17]. These PAMs can only modulate the activity of ORs when the orthosteric agonists occupy the receptor, indicating that PAMs maintain spatial and temporal control of receptor signaling in vivo. Endogenous opioid peptides are released at locations in the brain or spinal cord where they are required, namely, where pain occurs [18]. Therefore, the use of PAMs could provide drugs with improved adverse effect profiles or lesser tolerance and dependence than orthosteric opioid receptor agonists. Therefore, G protein-biased PAMs for ORs are also potential candidates for analgesics with fewer adverse effects.

Oxytocin (OT), composed of nine amino acids, is synthesized in the paraventricular nucleus (PVN) and supraoptic nucleus of the hypothalamus. OT is secreted from the posterior pituitary, which has a wide range of physiological functions, such as uterine contractions, maternal/social behavior, and antistress effects [19,20,21]. Some studies have shown that OT alleviates pain in several brain regions and the spinal cord, but the molecular mechanism remains unclear [22,23,24]. It is presumed that multiple pathways are involved in the analgesic mechanism of OT, namely, OT receptor-dependent and OT receptor-independent pathways. The OT receptor-dependent pathway is a hypothalamospinal oxytocinergic pathway. PVN stimulation or OT administration activates presynaptic OT receptors superficially located in the dorsal horn (laminae I and II) and subsequently excites inhibitory GABAergic interneurons [25,26,27,28]. Activation of GABAergic interneurons presynaptically inhibits nociceptive nerves (Aδ-fiber and C-fiber). In a study involving the OT receptor-independent pathway, OT-induced analgesia was absent in vasopressin-1A receptor null mutant mice in the radiant heat paw-withdrawal test and von Frey test [29]. OT also attenuates pain via desensitization of transient receptor potential vanilloid 1 (TRPV1) [30]. Moreover, the endogenous opioid system is involved in OT-induced analgesia. Injection of OT into the periaqueductal gray was found to increase endogenous opioids, and the OT-induced antinociception was antagonized by the OR antagonist naloxone [31,32]. In our previous study, we found that OT acted as a PAM and enhanced MOR activity [33]. Other groups found that OT-induced antinociception in rats was partially inhibited by the antagonists of MOR and KOR, but not of DOR [34,35]; these findings suggested that OT-induced analgesia may be mediated by KOR and MOR but is not mediated by DOR. However, the relationship between OT and DOR and KOR, such as that of agonists or PAMs, is not well understood.

In the current study, we investigated whether DOR and KOR act as PAMs, by using human embryonic kidney-293 (HEK293) cells stably expressing the human DOR or KOR. To this end, we performed CellKey^TM^ and cAMP assays for G protein signaling pathways and internalization assays for β-arrestin signaling pathways. In addition, we examined the binding affinity between OT and ORs by radioligand competitive OR-binding assay using Chinese hamster ovary (CHO) cells expressing human KOR.

## 2. Materials and Methods

### 2.1. Materials

The human OT, dynorphin A, and β-endorphin peptides were purchased from Peptide Institute Inc. (Osaka, Japan). Met-enkephalin and Leu-enkephalin were provided by the Department of Medicinal Chemistry, School of Pharmacy, Kitasato University (Tokyo, Japan). The remaining reagents were purchased from the indicated companies: (-)-U-50488 hydrochloride (U50488) from Tocris Bioscience (Bristol, UK); SNC80, forskolin, 3-isobutyl-1-methylxanthine (IBMX), and Ro 20-1724 from Sigma-Aldrich (St. Louis, MO, USA); morphine hydrochloride from Takeda Pharmaceutical Co., Ltd. (Tokyo, Japan); and fentanyl citrate injection solution from Janssen Pharmaceutical K. K. (Tokyo, Japan). Forskolin, IBMX, and Ro 20-1724 were diluted with DMSO, whereas the other reagents were diluted with H_2_O.

### 2.2. Establishment of HEK293 Cells Stably Expressing Human DOR or KOR

For human DOR clones, a combined fragment of the T7-fused tag and human DOR (NM_000911) was synthesized and amplified using the pCR^®^-Blunt II-TOPO^®^ vector (Life Technologies, Carlsbad, CA, USA). Subsequently, the combined fragment of T7-human DOR was transferred to pcDNA3.1-hygromycin (-) vectors (Life Technologies) with HindIII and EcoRV sites. The expression constructs were transfected into HEK293 cells (ATCC^®^, Manassas, VA, USA) by using X-tremeGENE HP DNA transfection reagent (Roche, Basel, Switzerland) according to the manufacturer’s instructions. At 48 h after transfection, the cells were cultured in culture medium containing 250 µg·mL^−1^ hygromycin B (Fujifilm Wako Pure Chemical Corporation, Osaka, Japan) to establish stably transduced cell lines. Monoclonal cells were selected on the basis of DOR activity measured using the CellKey^TM^ assay. Additionally, the cDNA of human KOR (NM_000912) inserted into the pFN21KSPc Halo Tag vector was amplified according to the manufacturer’s instructions (Kazusa DNA Research Institute, Chiba, Japan). HEK293 cells stably expressing human KOR were generated via transfection of both Halo-human KOR plasmids and pcDNA3.1-neomycin (-) plasmids (Invitrogen, Carlsbad, CA, USA) by using Lipofectamine 2000 (Invitrogen) and were selected using 700 µg·mL^−1^ G418 (Gibco, Carlsbad, USA) to establish stable expression. HEK293 cells stably expressing both human KOR and Glosensor^TM^ 22F protein were created by transfecting pGloSensor^TM^-22F plasmids (Promega, Madison, WI, USA) with a hygromycin B-resistant plasmid into HEK293 cells stably expressing human KOR.

### 2.3. Cell Cultures

HEK293 cells stably expressing human DOR, human KOR, or coexpressing both human KOR and Glosensor^TM^ 22F protein were maintained at 5% CO_2_ and 37 °C in Dulbecco’s modified Eagle’s medium (DMEM) high glucose (FUJIFILM) supplemented with 10% fetal bovine serum (Gibco) and penicillin (100 U·mL^−1^)/streptomycin (100 mg·mL^−1^) (Nacalai Tesque, Kyoto, Japan). To maintain DOR expression, KOR expression, and KOR and Glosensor^TM^ 22F protein coexpression, these cells were treated with 250 µg·mL^−1^ hygromycin B, 700 µg·mL^−1^ G418, and both 100 µg·mL^−1^ hygromycin B and 700 µg·mL^−1^ G418, respectively.

CHO cells stably expressing human KOR were purchased from ChanTest Corp. (Cleveland, OH, USA), and maintained at 5% CO_2_ and 37 °C in Ham’s F12 (Gibco) containing 10% FBS (Hyclone Laboratories, Inc., Logan, UT, USA), 1% nonessential amino acids solution (Gibco) and 400 µg·mL^−1^ G418 (Roche).

### 2.4. CellKey^TM^ Assay

DOR and KOR activities were measured using the CellKey^TM^ assay as described previously [33,36,37]. Briefly, HEK293 cells stably expressing human DOR or human KOR were plated in 96-well plates (poly-d-lysine-coated CellKey^TM^ microplate; Sigma-Aldrich) at 7.0 × 10^4^ or 6.0 × 10^4^ cells/well, respectively. The cells were incubated in 5% CO_2_ at 37 °C for 21-24 h. The cells were washed with CellKey^TM^ buffer composed of Hanks’ balanced salt solution (1.3 mM CaCl_2_, 0.41 mM MgSO_4_·6H_2_O, 0.49 mM MgCl_2_·7H_2_O, 5.4 mM KCl, 0.44 mM KH_2_PO_4_, 4.2 mM NaHCO_3_, 136.9 mM NaCl, 0.34 mM Na_2_HPO_4_, 5.6 mM d-glucose, and 20 mM HEPES, pH 7.4) supplemented with 0.1% bovine serum albumin, following which they were incubated at 27–28 °C for 30 min. Following baseline measurement for 5 min, the data were acquired as changes in the impedance of an induced extracellular current (ΔZiec) in each well every 10 s for 25 min after drug injection. The extent of change in ΔZiec was expressed as the value obtained after subtracting the minimum ΔZiec from the maximum ΔZiec after drug injection, and the values were normalized to the E_max_ value of each agonist or specific agonist (SNC80 for DOR and U50488 for KOR). Dose-response curves were calculated as the ∆Ziec of each agonist subtracted from that of the vehicle (0.1 % H_2_O).

### 2.5. GloSensor^TM^ cAMP Assay

Intracellular cAMP production was measured using the GloSensor^TM^ cAMP assay (Promega) as described previously [33,37]. Briefly, HEK293 cells stably coexpressing human KOR and Glosensor^TM^ 22F protein were seeded at 4.0 × 10^4^ cells/well in a BioCoat^TM^ poly-d-Lysine 96-well plate (Corning; Corning, NY, USA) and incubated in 5% CO_2_ at 37 °C for 24 h. The cells were washed with Hanks’ balanced salt solution and incubated with GloSensor^TM^ reagent (Promega) at 25 ± 3 °C for 2 h. The cells were treated with test compounds (vehicle, U50488, dynorphin A and morphine) diluted with Hanks’ balanced salt solution containing 50 µM IBMX and Ro 20-1724 for 10 min. Subsequently, 3.0 × 10^−6^ M forskolin was injected, and luminescence (RLU) was measured in each well every 2.5 min for 30 min by using a Synergy^TM^ H1 system (BioTek Instruments Inc., Winooski, VT, USA). The responses were expressed in terms of the area under the time-luminescence intensity curve (AUC) of each sample. The values were normalized to the response of vehicle at 30 min.

### 2.6. KOR Internalization Assay

To analyze KOR internalization, we used the membrane-impermeable Halotag^®^ pH sensor ligand, as previously described by Manabe et al. for MOR^37^. Briefly, HEK293 cells stably expressing human KOR-fused Halotag^®^ were seeded at 9.0 × 10^4^ cells/well in an eight-chambered coverglass (Thermo Fisher Scientific, Inc., Waltham, MA, USA) and incubated in 5% CO_2_ at 37 °C for 21–24 h. The cells were washed with Hanks’ balanced salt solution and treated with 0.5 µM Halotag^®^ pH Sensor Ligand (Promega) at 37 °C for 15 min. The cells were washed again with Hanks’ balanced salt solution and treated with 4 µg/mL Hoechst 33342 (Dojindo Laboratories, Kumamoto, Japan) at 37 °C for 10 min. Red spots were observed using a Leica TCS SP8 lightning confocal microscope with a 40× oil immersion lens (Leica, Wetzlar, Germany). After the baseline was measured for 10 min, images were obtained in each well every 10 min for 120 min after drug injection. The acquired images were analyzed for changes in the fluorescence intensity of total red spots/cells (the number of cells was defined by the number of nuclei as determined by Hoechst staining) with MetaMorph^®^ 7.7 (Molecular Devices) and were normalized to those before injection (% of before injection at 0 min).

### 2.7. Radioligand Competitive OR Binding Assay

The binding assay was performed as previously described [33]. In brief, membranes prepared from CHO cells expressing human KOR were incubated for 2 h at 25 °C with 1.0 nM [^3^H]diprenorphine (PerkinElmer, Norwalk, CT, USA), OT (10^−11^–10^−5^ M) alone, and dynorphin A or U50488 (10^−11^–10^−5^ M) in the absence or presence of OT (10^−6^ M) in 50 mM Tris-HCl buffer (5 mM MgCl_2_, 1 mM EGTA, pH 7.4). The assay was terminated by collecting membranes on Filtermat B filters (PerkinElmer) using a FilterMate^TM^ harvester (PerkinElmer), followed by washing with 50 mM Tris-HCl (pH 7.4) three times. The radioactivity was then measured using a MicroBeta scintillation counter (PerkinElmer). Nonspecific binding was determined using 10 µM nonradioactive diprenorphine. The curves were calculated using a one-site inhibition-binding model.

### 2.8. Statistical Analysis

All data are presented in terms of mean ± standard error of mean (SEM) values. Statistical analysis and dose response curve fittings were performed using GraphPad Prism 8 (GraphPad Software, San Diego, CA, USA). All data were compared using one- or two-way ANOVA, followed by Bonferroni’s test. Statistical significance was set at *p* < 0.05.

## 3. Results

### 3.1. OT Alone Did Not Change Impedance (ΔZiec) in HEK293 Cells Stably Expressing Either Human DOR or KOR in the CellKey^TM^ Assay

**Figure 1:**
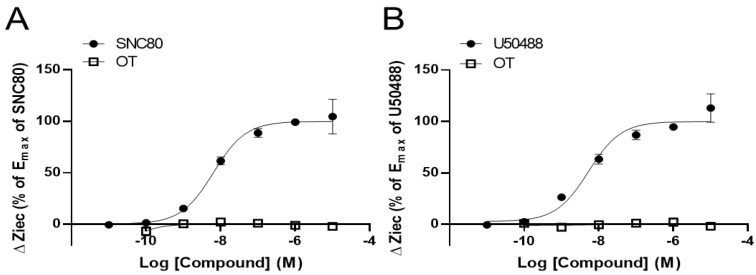
Oxytocin (OT) did not exert an agonistic effect on the human δ-opioid receptor (DOR) and κ-OR (KOR). HEK293 cells stably expressing DOR were treated with OT (10^−10^–10^−5^ M) or the selective DOR agonist SNC80 (10^−11^–10^−5^ M) (**A**). HEK293 cells stably expressing KOR were treated with OT (10^−10^–10^−5^ M) or the selective KOR agonist (-)-U50488H (U50488, 10^−11^–10^−5^ M) (**B**). The data were normalized by the E_max_ value of each selective agonist SNC80 (10^−5^ M; (**A**)) or U50488 (10^−5^ M; (**B**)) and are presented in terms of mean ± standard error of mean (SEM) values ((**A**); *n* = 3–4, (**B**); *n* = 3).

To clarify the effects of OT on DORs and KORs, we performed the CellKey^TM^ assay to examine whether OT exerts an agonistic effect on DORs and KORs using DOR- and KOR-expressing HEK293 cells, respectively. The selective DOR agonist SNC80 and the selective KOR agonist U50488 increased ΔZiec in DOR- and KOR-expressing cells, respectively, in a dose-dependent manner; OT (10^−10^–10^−5^ M) when used alone did not affect ΔZiec in both DOR- and KOR-expressing cells (Figure 1A,B).

### 3.2. OT Enhanced ΔZiec Induced by KOR Agonists but Not DOR Agonists in HEK293 Cells Stably Expressing Human KOR or DOR, Respectively

**Figure 2:**
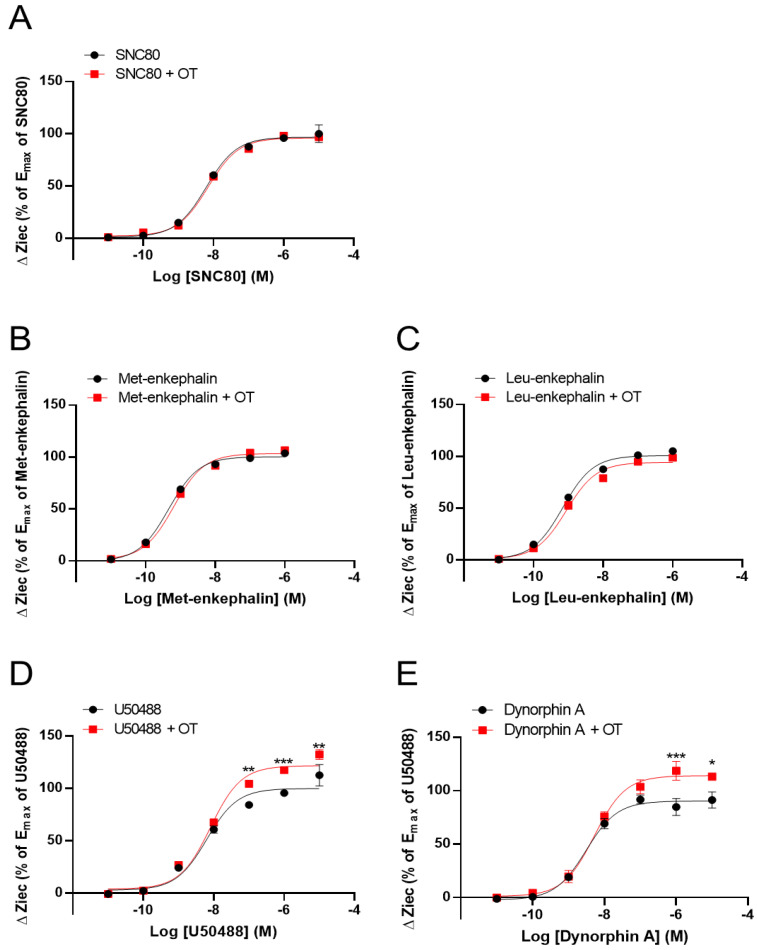
Effect of OT on the impedance (ΔZiec) induced by each KOR or DOR agonist in HEK293 cells stably expressing human KOR or DOR, respectively. Dose-response curves for SNC80- (**A**), Met-enkephalin- (**B**), and Leu-enkephalin- (**C**) induced ΔZiec in DOR-expressing cells, which were treated with or were not treated with 10^−6^ M OT. KOR-expressing cells were treated with U50488 (**D**) or dynorphin A (**E**) in the absence and presence of 10^−6^ M OT. The data were normalized by the E_max_ value of a selective DOR agonist (10^−5^ M SNC80) or a selective KOR agonist (10^−5^ M U50488) and are presented in terms of mean ± SEM values ((**A**); *n* = 5, (**B**); *n* = 3, (**C**); *n* = 3, (**D**); *n* = 4, (**E**); *n* = 3). Two-way ANOVA was performed, followed by Bonferroni’s test. * *p* < 0.05, ** *p* < 0.01, *** *p* < 0.001 compared to each agonist treatment alone.

We previously reported that OT acts as a PAM to enhance MOR activity [33]. Therefore, using the CellKey^TM^ assay, we tested whether OT had similar effects on DOR and KOR by using DOR- and KOR-expressing HEK293 cells, respectively. OT (10^−6^ M) did not affect DOR activity induced by the DOR agonists SNC80 (10^−11^–10^−5^ M) and Leu- and Met-enkephalin (10^−11^–10^−6^ M) (Figure 2A–C). In contrast, OT (10^−6^ M) enhanced the Ziec induced by the KOR agonists U50488 (10^−7^–10^−5^ M) and dynorphin A (10^−6^–10^−5^ M) (Figure 2D,E).

**Figure 3:**
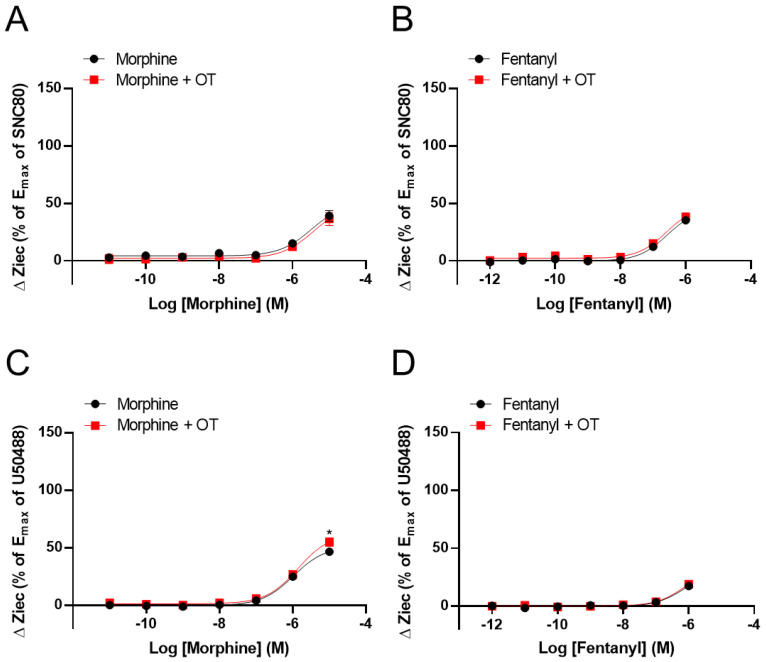
Effect of OT on ΔZiec induced by opioid analgesics in HEK293 cells stably expressing human DOR or KOR. Dose-response curves for morphine- (**A**) and fentanyl- (**B**) induced ΔZiec in the absence and presence of 10^−6^ M OT in DOR-expressing cells. KOR-expressing cells were treated with morphine (**C**) or fentanyl (**D**) in the absence and presence of 10^−6^ M OT. The data were normalized by the E_max_ value of a selective DOR agonist (10^−5^ M SNC80) or a selective KOR agonist (10^−5^ M U50488) and are presented in terms of mean ± SEM values (**A**); *n* = 4, (**B**); *n* = 3, (**C**); *n* = 4, (**D**); *n* = 4). Two-way ANOVA was performed, followed by Bonferroni’s test. * *p* < 0.05 compared to each agonist treatment alone.

We next investigated the effect of OT on ΔZiec induced by opioid analgesics that selectively activate MORs over DORs or KORs. Morphine and fentanyl partially increased ΔZiec in both DOR- and KOR-expressing cells, indicating that these drugs were partial agonists for both DOR and KOR (Figure 3A–D). In DOR-expressing cells, OT (10^−6^ M) did not enhance ΔZiec induced by morphine (10^−11^–10^−5^ M) and fentanyl (10^−12^–10^−6^ M) (Figure 3A,B). In contrast, in KOR-expressing cells, the ΔZiec induced by morphine (10^−5^ M) but not fentanyl (10^−12^–10^−6^ M) was slightly enhanced by OT (Figure 3C,D). OT increased the E_max_ values of U50488 and dynorphin A (Table 1) and also increased the maximum morphine response (Figure 3C) without affecting the LogEC_50_ values in KOR-expressing cells (Table 1).

**Figure 4:**
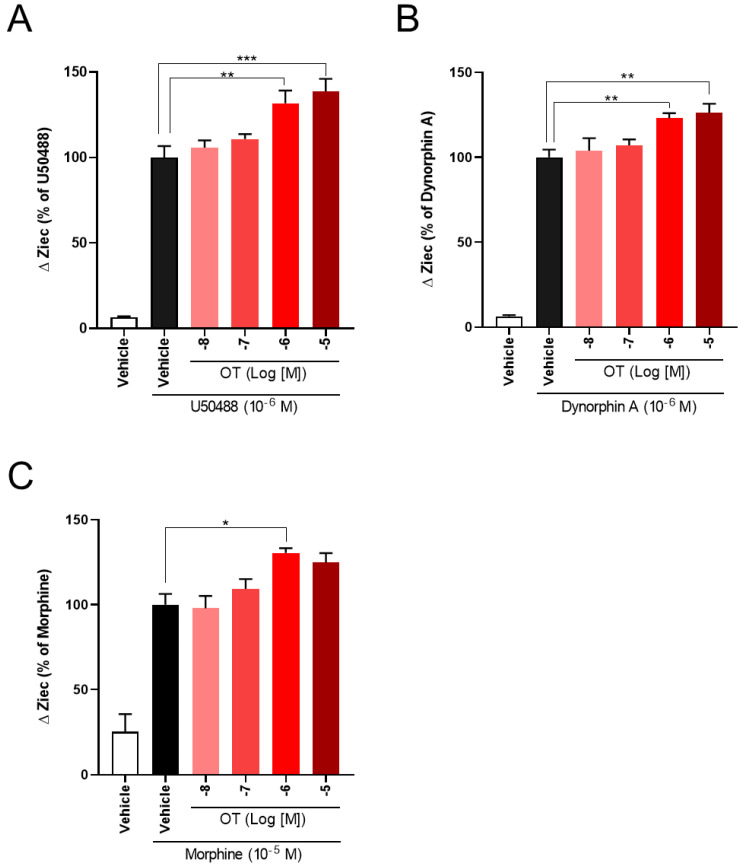
OT enhanced ΔZiec induced by KOR agonists in a concentration-dependent manner in HEK293 cells stably expressing human KOR. The cells were treated with 10^−6^ M U50488 (**A**), 10^−6^ M dynorphin A (**B**), or 10^−5^ M morphine (**C**). The data are presented in terms of mean ± SEM values ((**A**); *n* = 4, (**B**); *n* = 5, (**C**); *n* = 3–4). One-way ANOVA was performed, followed by Bonferroni’s test, * *p* < 0.05, ** *p* < 0.01, *** *p* < 0.001 compared to each agonist alone.

We examined the effects of OT concentration dependency on KOR activity in KOR-expressing cells. OT enhanced the increase in ΔZiec induced by U50488 (10^−6^ M), dynorphin A (10^−6^ M), and morphine (10^−5^ M) in a concentration-dependent manner (Figure 4A–C).

### 3.3. OT Enhanced Inhibition of Intracellular cAMP Induced by KOR Agonists in HEK293 Cells Stably Coexpressing Both Human KOR and Glosensor 22F Protein in the Glosensor^TM^ cAMP Assay

**Figure 5:**
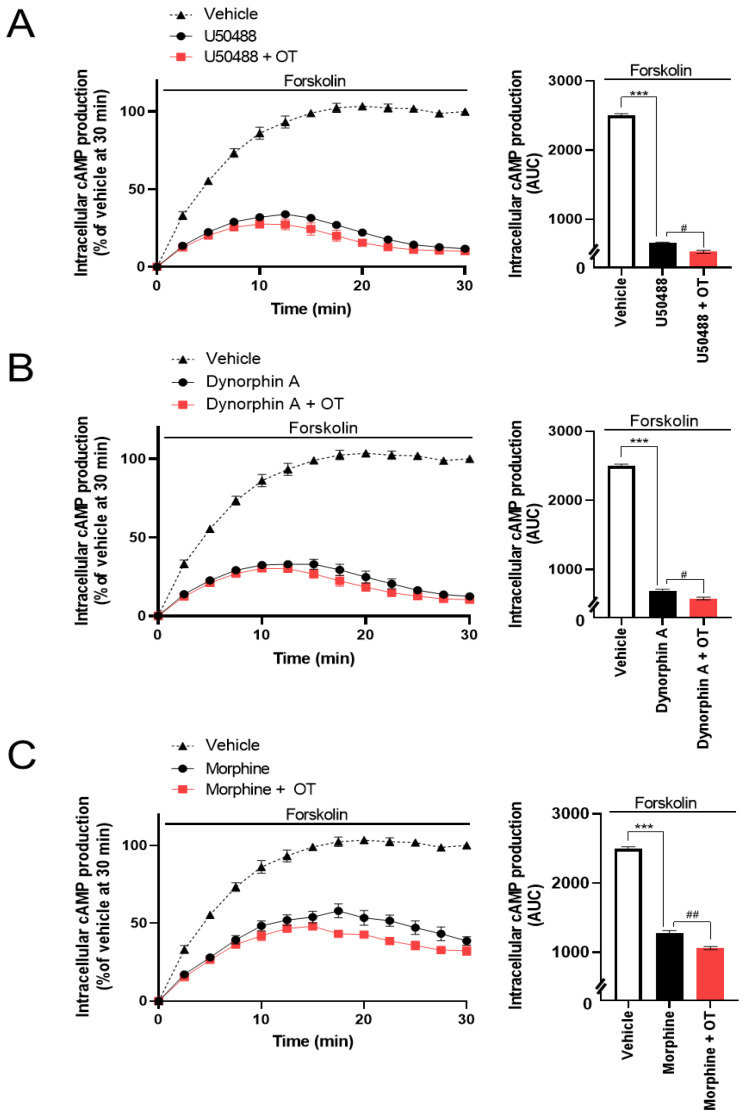
Effects of OT on inhibition of intracellular cAMP production induced by each KOR agonist in HEK293 cells stably coexpressing human KOR and Glosensor 22F protein. The cells were pretreated with 10^−6^ M U50488 (**A**), 10^−6^ dynorphin A (**B**), or 10^−5^ M of morphine (**C**) in the absence and presence of 10^−6^ M OT for 10 min and treated with forskolin (3 µM). The data are presented in terms of mean ± SEM values ((**A**); *n* = 3, (**B**); *n* = 3, (**C**); *n* = 3). Two-way ANOVA was performed, followed by Bonferroni’s test, *** *p* < 0.001 compared to vehicle. ^#^ *p* < 0.05 and ^##^ *p* < 0.01 compared to each agonist alone.

The Cellkey^TM^ assay detects the activation of GPCRs as whole-cell responses on the basis of changes in electrical impedance (ΔZ) [36]. Two of the most important GPCR signaling pathways are the G protein-dependent signaling and the β-arrestin-dependent signaling pathway. DOR and KOR are Gi/o protein-coupled GPCRs; therefore, their activation leads to the inhibition of adenylyl cyclase and decreased intracellular cAMP. Thus, we analyzed the effects of OT on KOR-mediated downstream signaling using the Glosensor^TM^ cAMP assay. U50488 (10^−6^ M), dynorphin A (10^−6^ M), and morphine (10^−5^ M) considerably suppressed intracellular cAMP production induced by forskolin (vehicle), adenylyl cyclase activator [38], and the inhibition was enhanced by treatment with 10^−6^ M OT (Figure 5A–C).

### 3.4. OT Did Not Potentiate the KOR Internalization Induced by KOR Agonists in HEK293 Cells Stably Expressing the Human KOR Fused Halotag^®^ in the Internalization Assay

**Figure 6:**
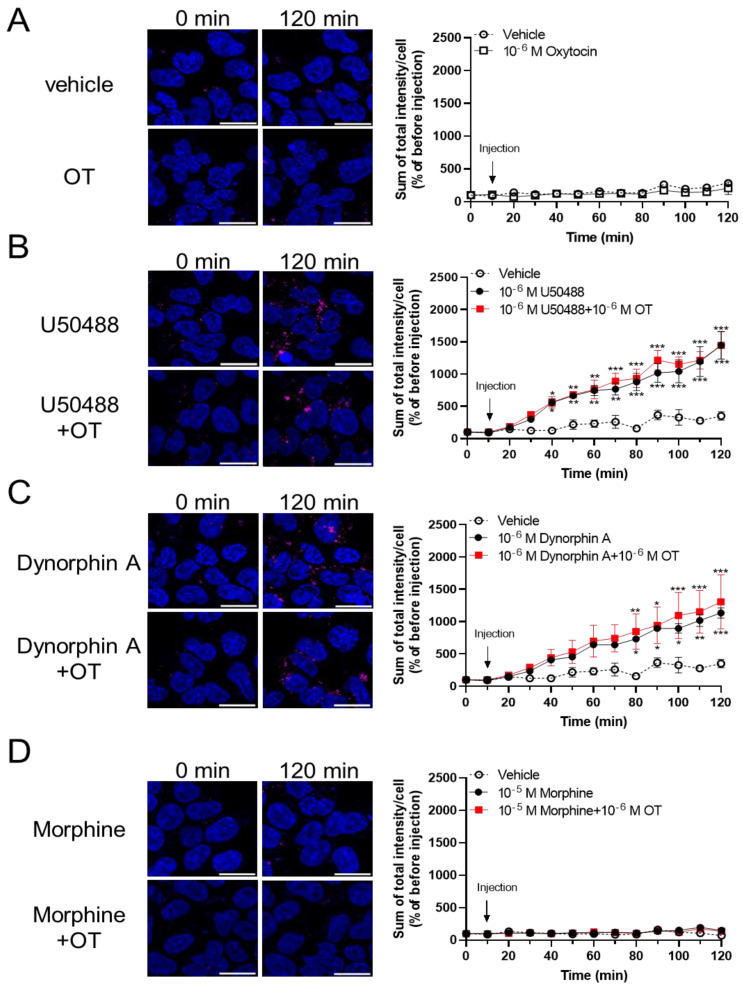
OT did not enhance internalization of KOR induced by dynorphin A and the U50488 agonist in HEK293 cells stably expressing HaloTag^®^-fused human KOR. Cells were dyed with a 0.5 mM pH sensor ligand and Hoechst 33342. After washing, the cells were treated with vehicle (**A**), U50488 (10^−5^ M), (**B**), dynorphin A (10^−6^ M) (**C**), or morphine (10^−5^ M) (**D**) in the absence and presence of 10^−6^ M OT. Time-response curves were calculated as the sum of total red fluorescence intensity per cell and were normalized to those before injection (at 0 min). The data are presented in terms of mean ± SEM values ((**A**); *n* = 4, (**B**); *n* = 3, (**C**); *n* = 3, (**D**); *n* = 3). Two-way ANOVA was performed, followed by Bonferroni’s test, * *p* < 0.05, ** *p* < 0.01, *** *p* < 0.001 compared to vehicle. Scale bar = 20 µm.

To assess the effects of OT on β-arrestin-dependent signaling mediated by KOR, we performed an internalization assay with the Halotag^®^ pH Sensor Ligand to quantify KOR internalization, as previously reported by Manabe et al. for MOR^37^. In this internalization assay, Halotag^®^-fused KOR bound to Halotag^®^ pH Sensor Ligand in an acidic environment (e.g., endosomes), fluoresced as red spots. Treatment with OT alone for 120 min did not increase the quantity of red spots (Figure 6A). U50488 (10^−6^ M) and dynorphin A (10^−6^ M) caused KOR internalization; however, very few red spots were noted on treatment with morphine (10^−5^ M), until 120 min after drug injection (Figure 6B–D). In contrast to the cAMP assay findings, OT did not significantly affect these phenomena in this assay (Figure 6B–D).

### 3.5. OT Did Not Affect [^3^H]Diprenorphine Binding to CHO Cells Stably Expressing Human KOR in the Radioligand Competitive OR Binding Assay

**Figure 7:**
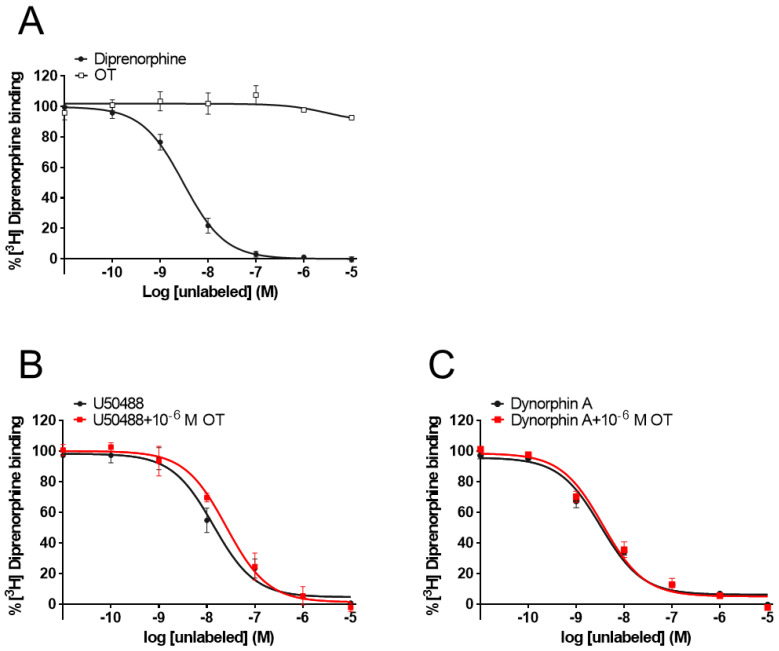
OT neither bound to orthosteric binding sites nor enhanced the binding affinity of KOR agonists for KOR in membranes prepared from CHO cells stably expressing human KOR. Membranes prepared from KOR-expressing cells were treated with OT or dynorphin A alone (**A**). U50488 (10^−11^–10^−5^ M, (**B**) or dynorphin A (10^−11^–10^−5^ M, (**C**) were treated with or without 10^−6^ M OT. The data are presented in terms of the mean ± SEM values ((**A**); *n* = 3, (**B**); *n* = 3, (**C**); *n* = 3).

While some PAMs potentiate the binding affinity of agonists for the receptors [39,40], OT did not affect the binding affinity of MOR [33]. To determine whether OT binds to the KOR orthosteric binding sites or enhances the binding affinity of KOR agonists for KOR, we performed a radioligand competitive OR binding assay with [^3^H]diprenorphine. OT, when used alone (10^−11^–10^−5^ M), did not inhibit [^3^H]diprenorphine binding to KOR (Figure 7A). Furthermore, there was no difference in the LogIC_50_ and LogKi values between dynorphin A and dynorphin A+OT, or U50488 and U50488+OT, respectively (Figure 7B,C, Table 2).

## 4. Discussion

To our knowledge, the current study is the first to indicate that OT facilitates both an increase in ΔZiec and decrease in intracellular cAMP induced by KOR agonists without affecting KOR internalization induced by these agonists. However, the signaling pathways induced by DOR were unaffected by OT. In addition, OT did not affect the binding affinity of KOR agonists for KOR. Taken together, these data suggest that OT induces G protein-biased allosteric modulation of KOR without affecting the binding affinity of KOR agonists.

Two of the most important GPCR signaling pathways are the G protein-dependent signaling and the β-arrestin-dependent signaling pathways [7,8]. Since the CellKey^TM^ assay results were similar to the cAMP assay results [33,37], the CellKey^TM^ assay was expected to primarily detect G protein-mediated signaling pathways [36]. In the current study, OT did not increase ΔZiec in cells expressing DOR or KOR and did not inhibit the binding of [^3^H]diprenorphine to KOR, which indicates that OT does not act as an agonist on DOR or KOR. Similar to this study, our previous study showed that OT did not act as an agonist of MOR, suggesting that OT did not directly affect the orthosteric binding sites of ORs [33].

The magnitude of the effect of PAMs on receptor signaling depends on two factors: the ability to change the binding affinity of the receptor to an orthosteric agonist (α) and intracellular responses of orthosteric agonists (β), as defined by Leach et al. [41,42]. The effect of PAMs can be classified into three patterns on the basis of α and β factors: both α and β, only α, and only β. In the current study, OT did not enhance the binding affinity of U50488 and dynorphin A to KOR, whereas it significantly enhanced the E_max_ of these agonists, as determined by the CellKey^TM^ assay. Hence, OT can be classified as type β, which changes the intracellular responses of orthosteric agonists.

Similar to the findings for KOR, we previously reported that OT is a type β PAM of MOR^33^. The reason why OT altered the activities of both MOR and KOR but not DOR is an area for future study. BMS-986187 was reported as a PAM for DOR in 2015^17^, but it was demonstrated to be a PAM for MOR and KOR [43]. In addition, BMS-986122, a PAM for MOR, was also a silent allosteric modulator (SAM) for DOR, indicating that it is an allosteric antagonist for DOR. Molecular docking studies revealed that BMS-986122 and BMS-986187 have the potential to bind to the same allosteric binding sites in transmembrane domains 2 and 7 on ORs [44,45,46,47], suggesting that these domains are important for the allosteric ligand binding site on the MOR, DOR, and KOR. Further studies are required to investigate which amino acids are involved in selectivity of PAM for OR subtypes.

In clinical practice, opioid analgesics are commonly used for managing cancer pain. Opioid analgesics mainly activate MOR but also partially activate DOR and KOR. Therefore, we investigated the effects of OT on the DOR and KOR activities induced by morphine and fentanyl. In our CellKey^TM^ and GloSensor^TM^ cAMP assays, OT potentiated the morphine-induced activation of KOR but not DOR, although the maximum responses of morphine for DOR and KOR were almost the same (DOR: 50.6% ± 7.01%; KOR: 51.5% ± 2.06%). In contrast, OT did not enhance fentanyl-induced activation of the DOR or KOR. The differences in these results may arise for two reasons: One possibility is that the level of KOR activity induced by fentanyl did not reach the extent to which OT would behave as a PAM for KOR; while the maximum response of morphine for KOR reached approximately 50% against the full KOR agonist U50488 (the maximum response of fentanyl for KOR was approximately 30%). Second, the PAM activities of OT for KOR were too weak to affect the fentanyl-induced conformational changes in KOR. The effect of OT on fentanyl-induced MOR activity was lesser than that on morphine-induced activity [33]. Thus, overall, the findings suggest that OT acts as a selective KOR-PAM; however, further studies are required for confirmation.

Several studies have implied that activation of β-arrestin-dependent signaling in KOR induces unfavorable effects, including sedation and dysphoria [48,49,50]. Since KOR internalization has been found to disappear in β-arrestin 2-knockout mice in primary striatal neurons [51], it is important to examine the effect of OT on KOR internalization in order to elucidate the effect of OT on KOR-mediated β-arrestin-dependent signaling. U50488- or dynorphin A-induced KOR internalization was not enhanced by OT, indicating that OT did not cause allosteric modulation of β-arrestin-dependent signaling of KOR. In GPCRs, including ORs, PAMs can cause biased modulation via conformational changes in receptors to elicit a specific intracellular signaling pathway [16,52]. Some groups have synthesized G protein-biased OR-PAMs [50,53]; the small molecule triazole 1.1 was found to show biased KOR agonism for G protein signaling over β-arrestin-2 recruitment in cell-based assays [50]. Taken together, these data suggest that OT is a G-protein-biased KOR-PAM.

We previously reported that OT behaves as a MOR PAM [33]; therefore, OT would be directly involved in signal pathways mediated by MOR and KOR. In addition, some studies have shown that the analgesic effect of OT injected into the periaqueductal gray, nucleus accumbens, and spinal cord was attenuated by injection of the MOR antagonist β-funaltrexamine and the KOR antagonist nor-binaltorphimine [34,35,54]. Hence, we propose that the PAM activity of OT for both MOR and KOR contributes to one of the analgesic mechanisms of OT.

To date, KOR agonists have not been developed as analgesics because of severe adverse effects such as dysphoria, hallucination, and sedation [55]. Recent studies revealed that injection of β-arrestin-2-knockout mice with KOR agonists evoked antinociceptive efficacy without these adverse effects [56,57]. Therefore, G protein-biased KOR agonists are expected to be novel analgesics with fewer adverse effects. KOR-PAMs are also candidates for novel analgesics because KOR-PAMs only modulate the activity of KOR when endogenous opioid peptides are released, namely, when there is pain. In addition, Brust et al. showed that the G protein-biased KOR-PAM triazole 1.1 induced antinociception without inducing sedation, decreasing dopamine concentrations, or producing behavioral indicators of dysphoria [50]. In the current study, we showed that OT might be a G protein-biased KOR-PAM, suggesting that OT is a candidate analgesic adjuvant without adverse effects. However, further in vivo experiments are required to investigate the influence OT has on adverse effects via KOR signaling.

One weakness of this study is that we did not analyze the interaction of oxytocinergic and opioidergic neuronal systems using animal pain models. Many studies have demonstrated that OT improves pain in several animal models [30,32,34,58]. In addition, some reports have shown that activation of the OT receptor upregulates prepro-enkephalin gene expression, which is the precursor for enkephalin, the endogenous ligand for MOR [59,60]. However, other studies have also reported that endogenous opioids decrease OT release through MOR and KOR mechanisms in response to physiological context (e.g., stress, pregnancy, etc.) [59,61,62,63]. In clinical studies, there is not sufficient evidence regarding the effects of OT on pain [64]. Therefore, further research is needed to evaluate the interaction of oxytocinergic and opioidergic neuronal systems and the effects of OT on pain using animal and clinical studies.

In conclusion, our current in vitro study indicated that OT has both MOR-PAM activity and KOR-PAM activity but does not have DOR-PAM activity. OT enhances G protein signaling without affecting β-arrestin signaling in KOR, indicating that OT has potential as a G protein-biased PAM of KOR. Thus, OT might act as a natural analgesic against pain via modulation of OR signaling.

## Figures and Tables

**Table 1:** LogEC_50_ and E_max_ of each opioid in the CellKey assay, in the absence and presence of 10^−^^6^ M OT in HEK293 cells stably expressing human DOR or KOR.

OR	Compounds	LogEC_50_ (M)		E_max_ (%)	
		Agonist	Agonist + OT	Agonist	Agonist + OT
DOR	SNC-80	−8.20 ± 0.11	−8.15 ± 0.06	100 ± 3.23	99.13 ± 1.77
	Met-enkephalin	−9.38 ± 0.08	−9.34 ± 0.11	107.8 ± 2.33	112.65 ± 3.43
	Leu-enkephalin	−9.23 ± 0.09	−9.04 ± 0.12	108.4 ± 2.71	103.1 ± 3.59
	Morphine	n.d.	n.d.	n.d.	n.d.
	Fentanyl	n.d.	n.d.	n.d.	n.d.
KOR	U50488	−8.20 ± 0.12	−8.09 ± 0.07	100.0 ± 3.47	121.7 ± 2.75 **
	Dynorphin A	−8.51 ± 0.11	−8.28 ± 0.09	90.3 ± 2.71	114.1 ± 2.97 **
	Morphine	n.d.	n.d.	n.d.	n.d.
	Fentanyl	n.d.	n.d.	n.d.	n.d.

LogEC_50_ and E_max_ are expressed in terms of mean ± SEM values (*n* = 3–5). Unpaired *t*-test was performed; ** *p* < 0.01 compared to each agonist treatment alone. n.d.: not determined.

**Table 2:** LogIC_50_ and LogKi of each opioid in radioligand competitive OR binding assay, in the absence and presence of 10^−6^ M OT in HEK293 cells stably expressing human KOR.

	LogIC_50_ (M)		LogKi (M)	
	Agonist	Agonist + OT	Agonist	Agonist + OT
Diprenorphine	−8.52 ± 0.06	n.m.	−8.86 ± 0.06	n.m.
U50488	−7.86 ± 0.11	−7.60 ± 0.12 ^n.s.^	−8.20 ± 0.11	−7.94 ± 0.12 ^n.s.^
Dynorphin A	−8.49 ± 0.09	−8.45 ± 0.09 ^n.s.^	−8.83 ± 0.09	−8.79 ± 0.09 ^n.s.^

LogIC_50_ and LogKi are expressed in terms of mean ± SEM values (*n* = 3). LogKi was calculated using one-site binding model (Fit Ki) by GraphPad Prism 8. Unpaired *t*-test was performed; n.s.; not significant, compared to each agonist treatment alone; n.m.: not measured.

## Data Availability

The data presented in this study are available in this article.

## Abbreviations

GPCR	G protein-coupled receptor
HEK293	human embryonic kidney 293
OR	opioid receptor
OT	oxytocin
PAM	positive allosteric modulator
TRPV1	transient receptor potential ankyrin 1
MOR	µ-opioid receptor
DOR	δ-opioid receptor
KOR	κ-opioid receptor
PAM	positive allosteric modulator
U50488H	(-)-U-50488 hydrochloride
PVN	paraventricular nucleus
IBMX	3-isobutyl-1-methylxanthine
DMEM	Dulbecco’s modified Eagle’s medium
ΔZiec	impedance of an induced extracellular current
AUC	area under the time-luminescence intensity curve
CHO	Chinese hamster ovary
n.d.	not detected
SEM	mean ± standard error of mean
